# Primary cesarean section rate among full-term pregnant women with non-previous uterine scar in a hospital of Vietnam

**DOI:** 10.1016/j.heliyon.2022.e12222

**Published:** 2022-12-09

**Authors:** Anh Duy Nguyen, Giang Thi Tra Duong, Dat Tuan Do, Duc Tai Nguyen, Duc Anh Tran, Thuong Thi Huyen Phan, Toan Khac Nguyen, Ha Thi Thu Nguyen

**Affiliations:** a Hanoi Obstetrics and Gynecology Hospital (HOGH), 100000 Hanoi, Viet Nam; b Hanoi Medical University (HMU), 100000 Hanoi, Viet Nam; c Vietnam National University, Hanoi-University of Medicine and Pharmacy (VNU Hanoi-UMP), 100000 Hanoi, Viet Nam

**Keywords:** Low-risk pregnancies, Primary cesarean section rates, Indications

## Abstract

**Aim:**

This article aims to determine the contributing indications for primary cesarean sections among full-term pregnant women with non-previous uterine scars and suggests several potential solutions to reduce the cesarean section rate.

**Methods:**

This is a descriptive study with data being retrospectively collected from electronic medical records (EMRs) at Hanoi Obstetrics & Gynecology Hospital, Vietnam, in 2020. We studied 23,631 women at ≥37 weeks of gestation with non-previous uterine scars. Main ICD-10 categories of diagnosis on the EMRs were used to classify the indications. The proportions of indications for primary cesarean sections were calculated, thereby offering potential solutions to reduce the cesarean section rate.

**Results:**

The proportion of cesarean sections among full-term pregnancies with non-previous uterine scars was 40.7%. The most common indications for primary cesarean sections were non-reassuring fetal heart rate tracing (40%), labor arrest (31%), and maternal request (11%). Among the low-risk pregnant women, the cesarean section rate was 35.9%, of which the percentages of labor arrest and non-reassuring fetal heart rate tracings and maternal request were 13.6%, 17.7%, and 4.6%, respectively.

**Conclusions:**

The proportion of primary cesarean sections among full-term pregnancies with non-previous uterine scars is high; non-reassuring fetal heart rate tracings, labor arrest, and maternal request were three main indications. It is necessary to build the strategies of health organizations regarding the management of clinical practices and the programs improving the knowledge, attitudes, practices of pregnant women and obstetricians regarding cesarean sections.

## 1 Introduction

Cesarean section (CS) is a life-saving intervention for mother and baby. However, the concern about a rapid increase in the cesarean section rate (CSR) is growing in all regions. The newest available statistic covering almost all births worldwide showed that the overall CSR was 21,1% [1]. By the year 2030, it is estimated that 28,5% of women will give birth through CS, with the lowest rate of 7.1% in sub-Saharan Africa and the highest of 63.4% in Eastern Asia [1]. Compared to vaginal delivery, an increase in the need for blood transfusion, the risk of anesthesia complications, infection, organ damage, and thromboembolic disease among women who underwent CS were observed [2]. In addition, a cesarean scar predisposes women to enormous gynecological issues such as pelvic adhesions, chronic pain, abnormal uterine bleeding, and infertility due to niche, as well as influences in subsequent pregnancies such as ectopic pregnancy, placenta accreta, placenta previa, and uterine rupture [2].

In a large hospital in the USA, in order of descending frequency, labor arrest, non-reassuring fetal heart rate tracings (NFHRT), fetal malpresentation, multiple gestations, and macrosomia were the most common indications for first-time CS [3]. The contributory factors to the rise in CSR were complex. Regardless of the changes in characteristics of the population influencing medical indications, such as an increase in obesity, multiple pregnancies, or older women, other non-clinical factors play an essential role in this increase [4].

In the East Asian and Pacific regions, where belief in spiritual elements more than modern science is prevalent, the CS population used more than doubled from 2000 to 2015, with the average rate of change of about 5% each year [5]. The rapid increase in CSR has also been observed in Vietnam, a Southeast Asia country, from 10% in 2002 to 28% in 2014, especially in urban areas (43%, 2014) [6]. In a recent study conducted in Danang city, located in an urban area in central Vietnam, CSR was extremely high, about 59%; and two prominent indications for primary CS were cephalopelvic disproportion and fetal distress [7]. According to the WHO, the optimal rate of CS is 10–15%, and the higher rate has not shown clear evidence of a decrease in maternal or fetal morbidity and mortality [4]. This equates to approximately four-fifths of CS cases in Vietnam being unnecessary, and instead being beneficial, could lead to harm to both mother and babies due to short-term and long-term complications [8]. This was more serious in the primary CS when the opinion “once a cesarean, always a cesarean” was still popular with the rate of repeat CS of about 90% [9]. Therefore, understanding the reasons for the primary CSR increase among full-term pregnant women is necessary to reduce overall CSR significantly.

In the global attempt to control CSR, this study aims to analyze the high rate of CS among full-term pregnant women with non-previous uterine scar (non-PUS) and propose an axis of improvement to lower the rate of CS in Vietnam, where the oriental culture heavily influences behaviors.

## 2 Materials and methods

### 2.1 Study design

A descriptive study was performed at Hanoi Obstetrics and Gynecology Hospital (HOGH), Vietnam from March to June 2021, and was approved by the Ethics Committee of HOGH with the Ethics No. IRB: 15/PS-HDDD. The data were retrospectively collected from the electronic medical records (EMRs) of pregnant women between 1st January 2020 and 31st December 2020.

### 2.2 Population and sample

**Figure 1:**
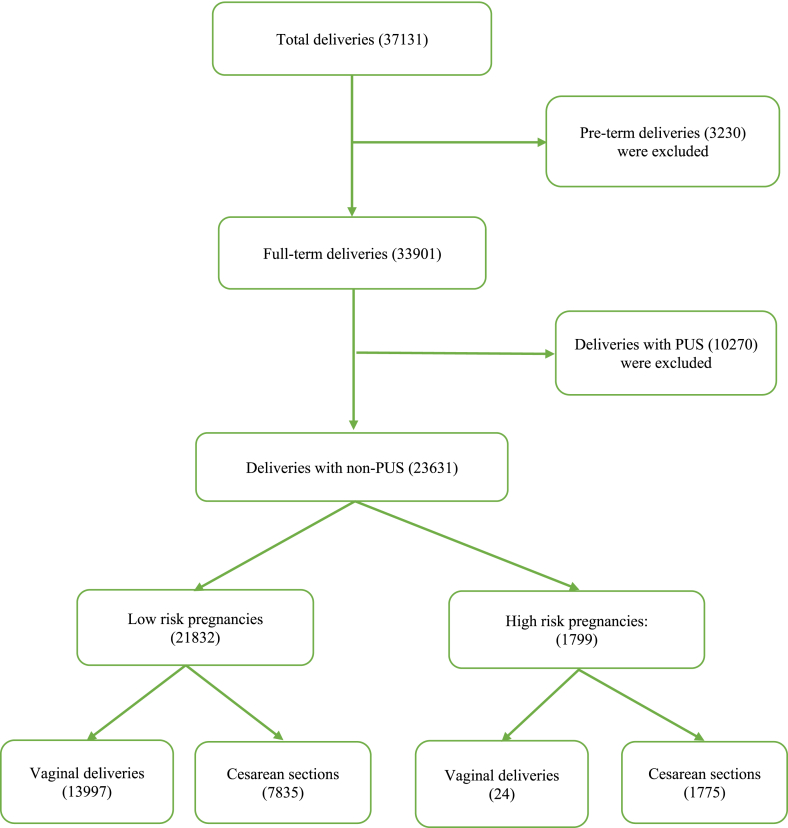
The flowchart.

In 2020, 37,131 women delivered at ≥22 weeks gestation, or the newborn weighed ≥500 g at HOGH. In this study, we investigated all 23,631 full-term pregnant women with non-PUS. Patients with pre-term pregnancies or PUS were excluded. The data collection progress was described step-by-step, as shown in Figure 1. All data were entered into Stata 14.0 for statistical analysis [10]. All variables were presented as frequencies and percentages.

### 2.3 Data collection

We collected the baseline characteristics of eligible women and their newborns, including delivery route, maternal age, number of fetuses, gestational age, birth weight, and infant gender.

We used the main ICD-10 categories of diagnosis in the EMRs made by clinicians when women were admitted to the hospital for deliveries. All women, diagnosed with one of the following six groups were classified as high-risk for pregnancies: multiple gestations, suspected macrosomia, malpresentation, pre-eclampsia, maternal/fetal conditions, and obstetrics mal conditions. The remaining women were classified as low-risk pregnancies. Suspected macrosomia was confirmed by the fetus’s estimated weight on ultrasound of more than 4000 g. Malpresentation consists of breech, face, and transverse presentation. Pre-eclampsia represents pre-eclampsia, eclampsia, and HELLP (Hemolysis, Elevated Liver enzymes, and Low Platelets) syndrome. Maternal/fetal conditions encompass maternal and/or fetal statuses, which predicted complications that might occur when delivering through the vagina, such as maternal cardiac diseases, maternal human immune deficiency virus infection, fetal anomalies, and intrauterine growth restrictions. Obstetric indications offer conditions related to the presence of the fetus intrauterine that require intervention to save the life of mothers and/or fetuses, such as placental abnormality, placental abruption, and cord prolapse. In both groups, low-risk and high-risk pregnancy, the participants were divided into subgroups, vaginal deliveries, and CS.

In the high-risk pregnancy group, there were six indications for primary CS: multiple gestations, macrosomia, malpresentation, pre-eclampsia, maternal/fetal indications, and obstetrics indications. There were three indications for primary CS in the low-risk pregnancy group: labor arrest, non-reassuring fetal heart tracings (NFHRT), and maternal request. The diagnosis of labor arrest and NFHRT during pregnancy and labor depends on the physician’s experiences according to local protocol. In our hospital, labor arrest was diagnosed when the change of cervical dilation in the active phase (>4 cm) was absent for more than 2 h with adequate uterine contractions. In local protocols, the indications of NFHRT included all women with category III tracing, partial women with category II tracing, and/or having meconium-stained amniotic fluid. This is in accordance with the FIGO consensus guidelines on intrapartum fetal monitoring CTG tracing classifications (2015). All Category III tracings were regarded as NFHRT and immediately indicated for CS. In the case of category II tracings, the decisions of continuously monitoring for labor, or performing cesarean depended on the clinical circumstances (null/multipara, stage of labor, decelerations/bradycardia), the results of utero resuscitation, and the physicians' experience. Additionally, some doctors regarded meconium-stained amniotic fluid as a sign of fetal distress and indicated CS because of NFHRT. In addition, we divided the maternal request for CS group into two subgroups, maternal request with social factors and maternal request with factors related to medical issues. The factors related to medical issues include preterm rupture of membrane, Insulin-treated diabetes, and post-term pregnancy.

In conclusion, there were nine main indications for primary CS: multiple gestations, macrosomia, malpresentation, pre-eclampsia, maternal/fetal indications, obstetrics indications, labor arrest, non-reassuring fetal heart tracings (NFHRT), and maternal request.

## 3 Results

Between January 2020 and December 2020, HOGH managed 23,631 women delivered at ≥37 weeks with non-PUS. The percentages of low-risk and high-risk pregnancies participants were 92,4% and 7,6% (21,832 and 1799/23,631), respectively. The proportion of CS among full-term pregnancies with non-PUS was 40,7% (9610/23,631). Of which, the percentage of CS due to NFHRT, labor arrest, and the maternal request were highest, at 16,4%, 12,5%, and 4,2% of all participants, respectively. Most women were more than 20 years of age (99,3%), and approximately three-fourths of the participants (75,3%) delivered at 39–40 weeks of gestational age. The number of women with multiple pregnancies was 528, accounting for 2,2% of all participants. The characteristics of full-term pregnant women with non-PUS and their newborns are detailed in Table 1.

**Table 1:** Characteristics of full-term pregnant women with non-PUS and their newborns.

Characteristics	Full-term pregnancies with non-PUS (n = 23,631)	
	Frequency (n)	Percentage (%)
**Delivery route**		
Cesarean sections	9610	40,7
*Multiple gestations*	513	2,2
*Macrosomia*	509	2,2
*Malpresentation*	314	1,3
*Pre-eclampsia*	93	0,5
*Maternal/Fetal indications*	139	0,5
*Obstetrics indications*	207	0,9
*Labor arrest*	2965	12,5
*Non-reassuring fetal heart rate tracings*	3875	16,4
*Maternal request*	995	4,2
Vaginal deliveries	14,021	59,3
***Age***		
<20	165	0,7
20–24	2930	12,4
25–29	10,682	45,2
30–34	6451	27,3
>35	3403	14,4
***Number of fetuses***		
Singleton	23,103	97,8
Multiples	528	2,2
***Gestational age (weeks)***		
37–38	5317	22,5
39–40	17,794	75,3
>41	520	2,2
***Birth weight (g)***		
<2500	662	2,7
2500–2999	5759	23,9
3000–3499	12,335	51,0
3500–4000	4891	20,3
>4000	497	2,1
***Infant gender***		
Girl	11,052	45,8
Boy	13,092	54,2

**Figure 2:**
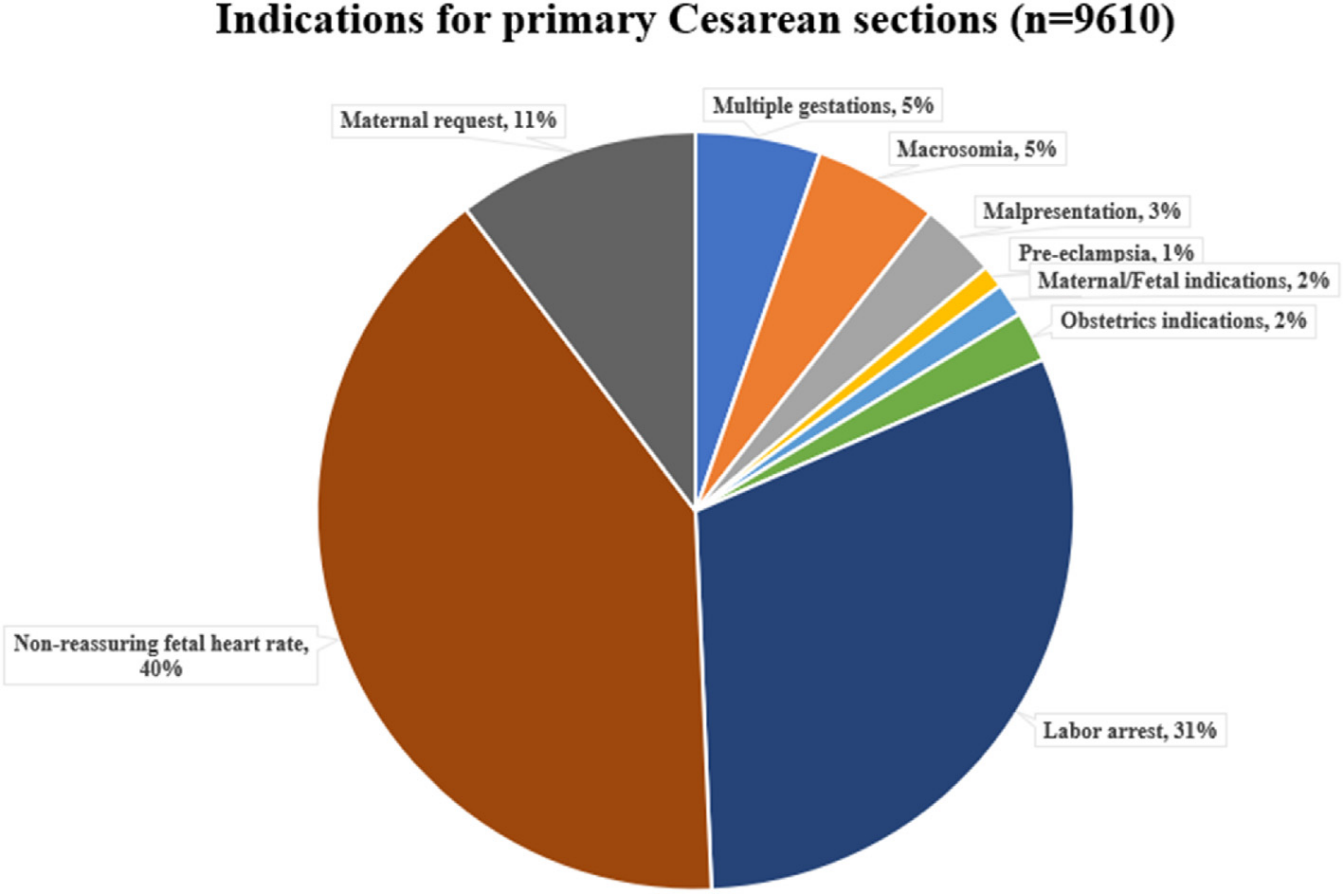
Contributing of indications for primary Cesarean sections.

Among 9610 women delivered via CS, the more significant part was low-risk pregnancy (82%), with three main indications: labor arrest (31%), NFHRT (40%), and maternal request (11%). Multiple gestations and suspected macrosomia were indicated in 10% of all cesarean deliveries. Only 1% of women with CS were pre-eclampsia (Figure 2).

The CSR of high-risk pregnant women was 98,7% (1775/1799). When macrosomia maternal/fetal/obstetric diseases were diagnosed among full-term women, the CS was performed immediately. Almost all women with multiple gestations (97,2%), malpresentation (98,7%), and pre-eclampsia (94,9%) were planned to end via cesarean deliveries in the study period. Within one year, there were 15 multiple pregnancies and four breech presentation women admitted to the hospital with a full-dilated cervix and who delivered via the vagina. Among low-risk pregnant women, the percentages of labor arrest and non-reassuring fetal heart rate tracings were 13,6% and 17,7%, respectively. Besides, 4,6% of women requested CS, and all were accommodated regardless of social factors or factors related to medical issues. The overall CSR of low-risk pregnancies was 35,9%. The detailed statistics are shown in Table 2.

**Table 2:** The Cesarean section rate of each indication group among full-term pregnant women with non-PUS.

Indication groups	Cesarean sections (n = 9610)	Vaginal deliveries (n =)
High risk Pregnancies (n = 1799)	1775 (98,7)	24 (1,3)
Multiple gestations (n = 528)	513 (97,2)	15 (2,8)
Macrosomia (n = 509)	509 (100)	0
Malpresentation (n = 318)	314 (98,7)	4 (1,3)
Pre-eclampsia (n = 98)	93 (94,9)	5 (5,1)
Maternal/Fetal indications	139 (100)	0 (0)
Obstetrics indications	207 (100)	0 (0)
***Low-risk pregnancies (n = 21,832)***	***7835 (35,9)***	***13,997 (64,1)***
High risk Pregnancies (n = 1799)	1775 (98,7)	24 (1,3)
*Non-reassuring fetal heart rate*	3875 (17,7)	
*Maternal request*	995 (4,6)	
Social factors	525 (2,4)	
Medical factors	470 (2,2)	
Values were given as no. (%)		

## 4 Discussion

The role of CS in reducing complications in high-risk pregnancies and abnormal labor was undeniable. However, CS, as with other surgeries, was accompanied by short-term and long-term complications. Uterine rupture, one of five severe obstetric complications, generally occurs in labor among women with PUS [11]. This leads to reluctance in a trial of a vaginal delivery setting and an increase in the rate of repeat CS. In addition, primary CSR has become a major driver of the overall CSR, accounting for approximately two-thirds of all CS in Vietnam [7]. Therefore, the efforts to reduce the primary CS are essential in lowering CSR strategies. Further, a global epidemiology survey performed by Ties Boerma et al. has shown that in Brazil and China, which had high CS rates, the largest contributor to overall CS were women with a gestational duration of at least 37 weeks (Robson’s group 1 to 5), about more than 80% in both countries [5]. Hence, in this study, we focused on pregnant women with more than 37 weeks of gestation and non-PUS.

In 2020, the proportion of primary CS among full-term pregnant women with non-PUS was 40,7%. It means two among five full-term pregnant women without PUS delivered through cesarean routes. This proportion in China, a country near and its culture is similar to Vietnam, was high as well, approximately 50% [12]. These statistics were alarming, with the proportion of primary CS in our hospital being approximately double that of many developed countries, particularly in western culture that is opposite to traditional oriental medical modalities such as Vietnam [13, 14]. The study of Boyle et al. performed in 228,562 deliveries at 23 weeks of gestation or greater in the USA, from 2002 to 2008, showed that the primary CSR was 21,3% [13]. In Northern Italy, the overall CSR was 24,2%, and the proportion of CS among women without previous cesarean scar was 19.6% [14]. Since 1985, WHO has stated that there was no justification for CSR higher than 10–15% of all deliveries [4]. Until now, it was true and has been confirmed by the population-based study of Ye et al. in 2014 [15]. This means that among 9610 CS of full-term pregnant women with non-PUS at Hanoi Obstetrics and Gynecology Hospital in 2020, an estimated 6073 (40.7–15 = 25.7%) to 7254 cesarean sections cases (40,7–10 = 30.7%) may have been unnecessary [16]. Our study showed that the proportion of labor arrest (31%), NFHRT (40%), and maternal request (11%) accounted for the majority (above 80%) of indications for primary CS. In addition, up to 35.9% of low-risk pregnancies ended via CS because of labor arrest, NFHRT, and maternal request; all the while, this proportion among US women was 12%, ranging from a low of 2.4% to a high of 36.4% [16]. Therefore, in this article, we mainly focus on discussing the reasons and then provide several potential solutions for three groups: labor arrest, NFHRT, and maternal request.

The number of CS due to labor arrest was 2965, accounting for 31% of primary CS. In contrast, in a study by Barber et al. (2013), the arrest of labor disorders contributed to 18% of primary CS in the US, a developed country with higher physician qualifications and more updated guidelines in medicine than Vietnam [17]. The difference in diagnosis of labor arrest may be blamed for the difference in CSR between our study and Barber. Although Friedman’s traditional definitions of labor arrest have been published for more than a half-century, it is still popularly used by most doctors in HOGH. According to this old criterion, labor is regarded as arrest when the changes of cervical dilation in the active phase (>4 cm) are absent for more than 2 h with a ruptured membrane and adequate uterine contractions (spontaneous or oxytocin augmentation) [3]. Some studies have revealed that the clinical practices based on Friedman’s criteria significantly rose CSR [3]. In 2008, a retrospective cohort study of 1014 low-risk women diagnosed with active phase arrest of labor, defined as Friedman’s definition, conducted by Henry et al., revealed that approximately one-third (33%) of women delivered vaginally without maternal or fetal complications [18]. Instead of cesarean deliveries, about 978 participants (33% of 2965) with diagnosed labor arrest in our study could deliver vaginally. In 2014, ACOG/SMFM announced a consensus guided that the active-phase arrest in the first stage of labor required the cesarean intervention should be diagnosed in women with cervical dilation of at least 6 cm, ruptured membranes, and failure to progress either 4 h of adequate uterine activity or at least 6 h of oxytocin administration [3]. Moreover, this consensus recommended that arrest second stage labor be diagnosed as the pushing duration of at least 2 h in multiparous women and at least 3 h in nulliparous. However, in our observation, most of our doctors agreed that CS should be indicated for women with a pushing duration of more than 1 h. They supposed that complications might take place with longer durations, despite a recent study showing that it is not true [19].

Regarding fetal conditions, cardiotocography was a useful tool used to monitor fetal health and was used widely for all women during labor in our hospital. However, several RCTs have determined that it has a false-positive value of 99.8% and is associated with a higher rate of CS [20]. The FHR category III are most justified indications for CS, while the fetal heart rate category II, an unresponsive fetus to external stimuli that may or may not be associated with fetal risk, could be an indication for CS due to NFHRT. Intrauterine resuscitative efforts and simultaneously finding the cause to improve the fetus’s condition with the FHR category II are recommended by experts [21]. However, because of the limitation in the evaluation of the variability of fetal heart rate as well as in resuscitation and finding the cause to ensure the fetus’s well-being in these cases, most of our physicians usually skip the two above steps and carry out the CS. This leads to the higher rate of CS because of NFHRT in our study, compared with the results of Barber et al. in 2013 (40% versus 32% of primary CS). Additionally, some doctors regarded meconium-stained amniotic fluid as a sign of fetal distress and as an indication for CS. This has been mentioned in some small studies; however, there is no clear evidence on the relation between meconium-stained amniotic fluid and fetal distress [22]. The main reason for the abuse of the NFHRT indications of our doctors was the fear of no guarantee of the well-being for fetuses because of the lack of their knowledge and skills in this field. The consequences of fetal loss for the doctors are extreme as relatives might sue the doctors and hospital.

The right of pregnant women to participate in the decision on the route of their deliveries is accepted widely by physicians, so maternal request is regarded as a primary CS indication, and is estimated to be about 2% of all CS [23]. This proportion in our study was double, at 4.2% of all participants and accounting for 11% of primary CS. In HOGH, as a woman requests primary CS, the physicians will discuss the complications of CS with her and her family. Because of the relative safety and the short-term benefits of CS and the ignoring intentional or unintentional of doctors about long-term complications during the discussion, most women had no change in their decisions. As in the same other regions, the reasons for choosing CS on request encompass convenience of scheduled birth; fear of the pain, process, length, and/or complications of labor and vaginal birth; prior poor labor experiences; concerns about fetal harm; concern about trauma to the pelvic floor and its sequelae; and need for control. We divided it into two subgroups in the maternal request group, including medical issues and social factors. In the former subgroup, some women experienced anxiety concerning the safety of their baby and feared something unfavorable happening, such as PROM, overdue pregnancy, or insulin-treated diabetes. Therefore, if doctors give insufficient information and an undetailed the delivery plan, they are willing to choose CS as it is the mother’s instinct to protect their baby. In the latter subgroup, the main social factor that influenced the decision for CS was that pregnant women and their families wanted to choose the day of delivery. Beyond convenience for arranging work, the date and time of birth are meaningful in the oriental culture, which is regarded as a crucial factor that influences the fate and life of each person. Thus, the maternal request for CS belongs to maternal choice and that of her family. Pregnant women and their families favor the baby’s benefits regardless of the risks for the mother. China, a large country in East Asia, has long suffered the harmful impact of this sort of culture, with the proportion of maternal request of all CS births up to approximately 25% [24]. This issue belongs to the sociology category, so to change, it needs the active and persistent participation of the entire society.

From the above observations in our hospital, we suggest several potential solutions to the problem of an increase in CSR. We focus on three main subjects: pregnant women, obstetricians, and hospital managers. It is essential to provide health education and support programs simultaneously with elevating the awareness among pregnant women about the risks and complications of cesarean, both short-term and long-term. General knowledge regarding vaginal and cesarean deliveries should be provided for all reproductive women, not only during pregnancy, but sooner such as in high school. For example, the day of birth could be scheduled via CS and vaginal deliveries by elective labor induction, and the pain in labor has not been an issue with epidural anesthesia. Health organizations need to build a system of diagnostic criteria based on the updated guidelines and strong evidence base, and simultaneously perform the seminar conferences or training programs to improve obstetricians' knowledge, attitudes, and skills in clinical practices. In addition, continuous peer evaluation and supervision of the physicians' knowledge and skills will ensure their clinical practices are up to current standards. Besides, as a bridge between patients and the health system, obstetricians need to be aware of the seriousness of increased CS and its consequences, thus having appropriate counseling for their patients.

The strength of our study is in using a large data pool in which to analyze results. The result is the ability to precisely present the disease pattern in HOGH, a large urban hospital in the capital of Vietnam. However, there are several limitations to this study. First, this data represents one hospital, and thus may not represent populations with different demographic and regional characteristics. Second, instead of using the Robson classification to access CS as guidance of WHO, we used the ICD-10 to categorize indications of groups of CS because ICD-10 is more common and is most used by doctors in our hospital. Third, because maternal and fetal medical records were separate, we could not collect data on fetal outcomes. Thus, there were no discussions about fetal outcomes in this study. To date, we have been building local protocols and applying them to clinical practice in our hospital. Consequently, we will evaluate the effectiveness of these solutions in reducing the high rate of C-sections in our hospital in the following study.

In conclusion, the proportion of primary CS among full-term pregnancies without PUS is high, about 40.7%, labor arrest, non-reassuring fetal heart rate tracings, and maternal request are three main indications for primary CS. The potential solutions include building the strategies of health organizations regarding the management of clinical practices and organizing programs to improve the knowledge, attitudes, and practices of both pregnant women and obstetricians regarding cesarean sections.

## Declarations

### Author contribution statement

Anh Duy Nguyen, Ha Thi Thu Nguyen: Conceived and designed the experiments; Performed the experiments; Analyzed and interpreted the data; Contributed reagents, materials, analysis tools or data; Wrote the paper.

Giang Thi Tra Duong: Conceived and designed the experiments; Analyzed and interpreted the data; Contributed reagents, materials, analysis tools or data; Wrote the paper.

Thuong Thi Huyen Phan: Conceived and designed the experiments; Contributed reagents, materials, analysis tools or data; Wrote the paper.

Toan Khac Nguyen: Conceived and designed the experiments; Analyzed and interpreted the data paper.

Dat Tuan Do, Duc Tai Nguyen, Duc Anh Tran: Performed the experiments.

### Funding statement

This research did not receive any specific grant from funding agencies in the public, commercial, or not-for-profit sectors.

### Data availability statement

Data will be made available on request.

### Declaration of interest’s statement

The authors declare no conflict of interest.

### Additional information

No additional information is available for this paper.
